# The Status of Chinese National Theoretical Discourse System and Its Correlation With Psychological Education of College Students

**DOI:** 10.3389/fpsyg.2021.755115

**Published:** 2021-12-24

**Authors:** Yueting Xiang, Yurong Miao, Jianxiang Zhang, Yujia Lin

**Affiliations:** ^1^School of Foreign Languages and Cultures, Xichang University, Xichang, China; ^2^Institute of Ethnic Literature, Yunnan Academy of Social Sciences, Kunming, China; ^3^Business School, Foshan University, Foshan, China; ^4^School of Journalism and Communication, Sichuan International Studies University, Chongqing, China

**Keywords:** national theory, discourse system, entrepreneurship psychological education, ideological and political class, Marxism

## Abstract

In recent years, in the face of new situations and problems in society, the discourse system, academic concepts, and related research perspectives of Chinese national theory have been strongly impacted by Western thought, making the discourse mode of western national theory become the second discourse system outside the traditional Chinese national theory. In order to further promote the construction of the Chinese national discourse system in college students' mental health education systems, the study first starts with the concept of national theory and discourse system and then uses the literature method to consult the development status of the Chinese national theoretical discourse system and the mental health education of college students. The results show that students' grade is directly proportional to the satisfaction of students with mental health education, that is, with the increase of grade, students are more and more satisfied with mental health education. The attendance rates of college students, high school students, junior middle school students, and primary school students are about 98, 87, 81, and 78%, respectively. In addition, students who are willing to actively accept psychological education account for about 38% of the total number of students, those who do not refuse account for 51% of the total number of students, and those who are unwilling to accept account for 11% of the total number of students. Among these students, their acceptance of mental health education will not be affected by other factors. Furthermore, the satisfaction score of college students with mental health education is about 4.1, the satisfaction score of high school students is about 3.6, the satisfaction score of junior middle school students is about 2.9, and the satisfaction score of primary school students is about 2.1. It reveals that the degree of satisfaction with mental health education is also related to grade. While taking mental health education courses, students not only realize a comprehensive understanding of the theory of the Chinese nation, but also greatly improve their national self-confidence and psychological quality. Moreover, it also strengthened by disseminating the theoretical discourse of the Chinese nation. Therefore, the exploration is of great significance to the development of the Chinese national theoretical discourse system in the psychological education of college students.

## Introduction

Nowadays, due to the accelerating process of globalization, countries in the world are not only competing fiercely in the economy, but also in politics. Discourse, as a symbol of rights and status of a country, is also an important form of national cultural communication. The establishment of the discourse system must be given enough attention (Qian et al., 2018). However, for a long time, China has been passively constrained by Western thinking and system, and the Chinese have been marginalized (Chen, 2019). As a result, China lacks discourse power in the world, and Chinese thinking has been ignored (Wu and Song, 2019). As an extension of ideology, education varies from country to country. There are essential differences between Chinese education and Western education (Rogoza et al., 2018; Wu et al., 2019). Chinese education should take the Chinese discourse system as the core, with distinct Chinese culture, Chinese characteristics, and Chinese style, which is not only the development requirement of Chinese pedagogy (Bowker, 2018) but also the development direction of Chinese education in the future. It can effectively promote Chinese education to go out of the country and go to the world stage, to make a strong voice in the world. Therefore, Chinese education must take the expression of the Chinese discourse system as the primary feature (Sofi and Najar, 2018), and show the Chinese national culture and way of thinking behind the Chinese language, to highlight the essential characteristics that are completely different from Western education. Only by establishing a sound discourse system, can China's local education enjoy a place in the process of academic internationalization (Danielle et al., 2018). Education is related to the development of a country and the prosperity of a nation, and the coordinated development of all ethnic groups is the basis for the progress of the whole society (Sheikh, 2018). In the process of building a modern power in an all-round way, China must solve the problems of ethnic discrimination, always adhere to the national theory, construct the discourse system of national theory, and open up the Chinese road with Chinese native language and theory, so as to tell Chinese stories, guide Chinese practice, establish the discourse system of national theory with Chinese characteristics (Wang, 2019), seize the priority discourse right in the international arena, and change Chinese state of aphasia. Therefore, how to establish a discourse system of national theory with Chinese characteristics and change the dilemma of Western thinking in Chinese education (Wang and Wang, 2019) is a problem that scholars need to study.

Any theory or viewpoint has its ideological roots. The analysis of the construction of the discourse system for the ideological and political education of college students from the perspective of cultural self-confidence also has its profound ideological roots. Cultural soft power, core values, all-round development of people, and educational discourse power are the theoretical support for the construction of ideological and political education discourse system for college students from the perspective of cultural self-confidence. It reveals that cultural self-confidence is closely related to the construction of a discourse system for the ideological and political education of college students (Shekhar and Huang, 2021). Gong (2018) has long proposed “establishing a socialist national theoretical system with Chinese characteristics.” In the article “keeping pace with the times and promoting the innovation of national theory,” it is mentioned that only by finding the laws of national problems and scientific interpretation, the national theory system of China can be created, its Chinese characteristics be highlighted, and the national theory be innovated (Gong, 2018). Peter (2019) defined and studied nationalism in the framework of ideological history. From this perspective, the research belongs to historical research. What it clarifies is how nationalism comes into being and the background characteristics of this theory (Peter, 2019). Qing (2019) believed that there are many problems in the discourse system of China. From the perspective of social science, the vast majority of philosophical and social science discourse systems are supported by Western theories, and there is no idea of building Chinese characteristic discourse system under the guidance of the theoretical system of China, which makes it difficult to have a strong voice on many international issues (Qing, 2019). German writer Lissgt (2020) focused on Habermas' understanding, criticism, and reconstruction of current German domestic politics and international politics in *Post National Structure*. In particular, the so-called global management system without a world government has aroused strong repercussions in contemporary Western political theorists and other practical political fields, and has even become a theoretical resource for the EU to carry out political reform. These scholars have expressed their views on the discourse of national theory from different angles (Lissgt, 2020).

At present, the study of the global discourse system just started, and the division of the human discourse system is also inconsistent. Chen divided the global discourse system into five regions: the Far East, the Middle East, the West, Latin America, and Africa, and then divided into 10 discourse systems: Chinese civilization, Japanese civilization, Indian civilization, Islamic civilization, Jewish civilization, American civilization, European civilization, Russian civilization, Latin American civilization, and African civilization. Obviously, this division is based on the traditional historical perspective (Chen, 2021). The modern global discourse system is not a simple addition to traditional history and civilization. Some scholars also divide human discourse into three levels: global, western, and eastern, or directly into two opposite categories: western and non-western. Such a division is inevitably general. At present, the world has formed a new comprehensive pattern composed of countries, nationalities, religions, international organizations, and regional factors. For example, some Asian countries, such as Japan, originally belong to the Asian civilization area and belong to the Asian discourse system in history, but at present, they are largely subordinate to the hegemonic discourse system led by the United States. Born in the Soviet Union, Ukraine originally belonged to the Siberian discourse system dominated by Russia, but now it has turned to the European discourse system. Therefore, the division of the global discourse system should be reconsidered from the perspectives of history and contemporary, reality and virtual, religion and civilization, region and global.

China has always advocated the concept of “harmonization culture,” whereas Western countries believe in hegemonism. With the rise of China, the West has never given up infiltrating into the ideological field of China and maintaining its hegemonic position in the world pattern by constantly rejecting and suppressing dissidents. Under the discourse impact of Western ideology, the discourse position of Chinese mainstream ideology has been impacted frequently, and once faced the danger of discourse aphasia. Based on this, China tried its best to revive the Chinese national culture. However, due to the pre-implantation of the western discourse system and the weak construction of the Chinese discourse system, especially the lack of awareness of ideological discourse power, the construction of ideological discourse system with Chinese characteristics has been difficult to obtain strong and solid cultural support; meanwhile, the discourse power of ideology in the virtual cultural space has been reset and decomposed, resulting in a serious impact on the guiding power of China's mainstream ideology (Wang, 2021).

This exploration aims to promote the construction of the discourse system of Chinese national theory, form an education system with Chinese characteristics in the process of adapting Marxism to China, and improve national self-confidence and recognition (He, 2019). The research innovation is to combine the discourse theory system of the Chinese nation with the political education of college students. Moreover, from the perspective of psychological education of college students, the ideological and political classroom with the construction of the national theory discourse system as the main guiding ideology is analyzed. Through consulting literature and data, questionnaire, and other methods of field research, the final conclusion obtained is as follows. The communication of the discourse system of national theory must take the college ideological and political class as the main way, which also plays a positive role in the mental health education of college students. Whether in theory or in practice, this exploration has a certain reference and guiding significance for the establishment and development of the discourse system of national theory.

Through the method of questionnaire, after understanding the theoretical discourse system of the Chinese nation and the current situation of college students' mental health education, the study analyzes the correlation of students' mental health education from multiple angles. Moreover, it is concluded that students' satisfaction with mental health education is not only related to the grade but also related to the attendance rate of the course. These conclusions and viewpoints are brand-new, which can be used for reference for subsequent related research. First, based on the concept of national theory and discourse system, the literature method is used to consult the development status of the Chinese national theory discourse system and the mental health education of college students. Then, the questionnaire method is adopted to study and analyze the correlation between the discourse system of the Chinese national theory and the mental health education of college students. Finally, the corresponding conclusions are drawn. Therefore, this exploration is of great significance to the development of the psychological education theory discourse system of college students.

## An Overview of the Discourse System of National Theory and the Psychological Education of College Students

### National Theory

“National theory” is an overview of ethnic issues based on socialism with Chinese characteristics. Under the background of the new era, the national theory mainly refers to the policy program with strategic guiding significance established with the core idea of adapting Marxism to China and the core content of national issues (Wei and Zhang, 2017). Discourse is an expression carrier of human emotion and wisdom (Li D., 2017), which can be divided into oral and written forms. Discourse system is the external form of discourse carrier, which is influenced by the knowledge, concept, and environment acquired by human beings, and constantly updated with the growth of values (Bai et al., 2018). Human wisdom must be expressed through discourse. Different expressions, namely different words and moods, have different influences on the crystallization of human wisdom under the surface of the discourse carrier. In order to study the construction of the discourse system of the Chinese national theory, not only does the national theory carried by the dialog system need to be innovated (Wang and Jin, 2018), but also the communication carrier needs to be expanded and improved.

A discourse system is a kind of (Wang, 2017) carrier with instrumental characteristics, which can carry thoughts, knowledge, ideology, and theoretical system. This carrier belongs to the external expression form of the discourse content under the surface of the discourse (Yang et al., 2020). The discourse system and ideology complement each other, and the types of ideology determine the types of discourse systems. Different discourse systems express and spread different values and interest demands. The discourse system is also a collection of civilization and culture. It is a part of a country's soft power and can represent the external image of a country. A good and complete discourse system can enable a country to fully display its national strength on the international stage and grasp its national discourse power (Jin, 2018).

Socialism with Chinese characteristics is the scientific connotation of the discourse system of Chinese national theory (Yang, 2017). First, the practical practice and development path of China are the realistic basis of the national theoretical discourse system of China. The discourse system can reflect the current development state of the country, present all kinds of problems in the development, point out the correct way to solve national problems and bring Chinese wisdom to the world. Furthermore, to construct the discourse system of the national theory of China is to show the world the propositions with Chinese characteristics, among which the “characteristics” include the Deng Xiaoping Theory, the important thought of Three Represents, the scientific outlook on development, and General Secretary Xi's new ideas and new strategies (Bai, 2018). These theoretical achievements, as Chinese characteristics, are the core of the discourse system of Chinese national theory. Moreover, Chinese people should have full confidence in the basic system and guiding ideology of China, and use the system and ideology to construct the discourse system of Chinese national theory. Finally, the excellent traditional culture of the Chinese is the internal gene of the Chinese national theoretical discourse system, and the cultural self-confidence is the guiding light to build a national theoretical discourse system with real Chinese characteristics and Chinese style (Wang, 2020).

The road of socialist development needs to hold high the great banner of Marxism to resist the ideological infiltration of foreign hostile forces and oppose Western discourse hegemony. The construction of the discourse system of national theory based on the national conditions of China should be under the guidance of the Marxist theoretical system. From the ideological point of view, Marxist national theory and the Western bourgeois national theory are two opposite theories (Li Z., 2017). The discourse system of the Western national theory serves the interests of capitalist regime and class and will sacrifice and exploit the interests of other nations, which has the narrow-mindedness of the bourgeoisie. There are still national oppression and exploitation in the contemporary capitalist world. In addition, “it shows more rule-based domination and exploitation of the vulnerable national groups, and the class oppression relationship in it has the characteristics of concealment and legalization.” When dealing with national issues, Marxist national theory considers not only the fundamental interests of the people of its own country but also the interests of the people of all national groups in the world, which has fundamental advantages. Therefore, China must adhere to the theoretical self-confidence of Marxism.

The ideological line of seeking truth from facts of the Communist Party of China is a complete theoretical system gradually formed, established, and developed with the process of the Chinese revolution, construction, and reform. It reflects the great process of the party's hard struggle for a century and moving toward success and maturity. The exploration process of the ideological line of seeking truth from facts is also the core idea of the sinicization of Marxism. The transformation of the discourse system of Marxist theory education in colleges is to take the theory close to reality, close to life, and close to students as the basic principle, fully consider the knowledge structure and thinking mode of contemporary college students, and explain and understand the profound theory with living examples in real life of college students, plain language, and a receptive way, so as to college students easy to understand and willing to accept the Marxist theory, and achieve the purpose of internalization in mind and externalization in practice. Strengthening the research on the discourse transformation of Marxist theory education in colleges can not only expand the basic path of the popularization of Marxism but also have crucial guiding significance for improving the teaching effect of ideological and political theory courses in colleges and cultivating qualified socialist successors (Woody and Arnold, 2017).

### Research Methods Used

(1) Literature method is a method to understand and prove the research object by consulting literature. Accumulating documents can save the documents completely, or collect the parts related to their own research topics by making cards, writing reading notes, and notes. (2) Comparison method, also known as the comparative analysis method, is an analysis method to prompt the difference between the actual number and the base through the comparison between the actual number and the base, to understand the achievements and problems of economic activities. Comparative analysis is often used in scientific inquiry activities, which is similar to the equivalent substitution method. (3) Questionnaire method is a widely used method in the social survey. It refers to the form used for statistics and investigation to express questions by raising questions. Researchers use this controlled measurement to measure the research problems and to collect reliable data. Among the three research methods, the questionnaire method is adopted, which is more suitable for the theme of this exploration. Moreover, it is more scientific to explore the correlation between the current situation of the theoretical discourse system of the Chinese nation and the psychological education of college students from the perspectives of grade and attendance.

### The Meaning of Ideological and Political Education Discourse

The discourse of ideological and political education is the mainstream ideology based on social ideology. On the basis of observing some language norms, ideological and political work, and organizing ideological and political education activities, its political quality and ideological and moral level have been improved. Ideological and political education in colleges mainly undertakes ideological and political theories. Teachers are engaged in the educational management services of students, but all teachers should shoulder the educational mission. Through the discourse of ideological and political education, they will pass on the ideological concepts and morality that meet the social requirements to college students, so that college students should not only understand, but also recognize these ideas and norms, externalize them into practical behavior, and then realize the educational objectives.

Ideological and political education is the “soft” management that changes the ideological concept of people and behavior under the influence of public opinion or moral norms. They complement each other through legal and administrative means and jointly play the role of managing society. The management function of the ideological and political education of college students has been very dramatic, which shows that the ideological and political education activities have a physical effect, have a substantive impact on the world outlook, life, and values of college students, and even slightly change their daily behavior, helping college students become red and dedicated socialist successors. It holds that the language of political education is directly related to this reality, but in order to use this word and give play to the way of thinking about the discourse of political education, teachers must grasp and understand the characteristics of the discourse of political education.

### Current Situation of the Psychological Education of College Students

The definition of psychological education is to use the basic principles of psychology and psychological technology to carry out education for the development of students, add psychological elements in traditional education, help students grow and develop in an all-round way, and cultivate the good psychological quality of individuals to make them form a harmonious personality. Different from mental health education and ideological and political education, it is a kind of development education, happiness education, and education that publicizes human nature (Gu, 2020).

Mental health refers to that individuals who can maintain a good and stable psychological state under the influence of different environments and factors and reasonably adjust and improve their internal psychological structure in constant contact with the external environment, so as to achieve coordination and harmony with the environment; moreover, in this process, individuals can gradually improve the function of psychological development and improve their personality. Mental health can be understood as a subjective psychological experience, and it is also a relative and changeable psychological state. As long as the inner state of an individual is stable and can be adjusted in a way of universal recognition in different environments, it can be called mental health. Although different scholars have had different views on the connotation of mental health for many years, most scholars have the same ideological purpose. They all believe that mental health is a kind of harmonious, good, and stable psychological state (Zochil and Thorsteinsson, 2018).

Generally, mental health education refers to, under the guidance of scientific theory, following the basic law of psychological activities for people, mobilizing all positive factors inside and outside through a variety of educational methods and means (Jin, 2017), cultivating good psychological quality in a planned and purposeful way, stimulating psychological potential, maintaining mental health, and taking the purpose of cultivating sound body and mind and perfect personality. Mental health education is a constantly developing educational concept, and it is also a kind of educational mode, which is constantly improved in practice, so it has a rich connotation. From this point of view, mental health education can be summarized from three aspects. First, mental health education is mainly aimed at the counseling and adjustment of psychological diseases and psychological obstacles; second, it is the guidance and help for the psychological conflict and confusion; third, it is the cultivation of good psychological quality and the development of psychological potential, so as to promote the health and all-round development of people. Generally, mental health education is not carried out separately for individuals with psychological barriers. It is for all people to maintain mental health, cultivate and improve sound personality and good personality psychological quality (Zhang, 2017), improve social adaptability, support, and promote the free and all-round development of people, which is the basic connotation of mental health education.

### National Theory: An Important Carrier of Mental Health Education of College Students

Education is a basic form of culture, and culture is an important carrier of education. From the macro point of view, the Marxist theory and the theoretical system of socialism with Chinese characteristics, which are conveyed in the ideological and political classroom, are part of the cultural system with Chinese characteristics. They carry rich contents of mental health education and influence the thoughts, psychology, and behaviors of people invisibly. Therefore, the ideological and political classroom is an important carrier for college students to learn national theory. Its educational function is to exert a subtle influence and control on the ideological beliefs of college students and behavior patterns through various ways and means, with its own thoughts as the basic educational content, so as to make students understand themselves correctly, strengthen their ability of psychological control and environmental adaptation, and make them achieve the harmony of body and mind. Moreover, it has always maintained relatively stable overall characteristics, in the process of integrating the lives of students, constantly dominating their thoughts and standardizing their behaviors (Yuan and Tang, 2019).

On the whole, as an important carrier of college students' mental health education, the basic elements of the ideological and political classroom itself are also permeated with many factors conducive to mental health education, which also make it an important means of mental health education in college students. Since its formation, it has gathered various factors and existed in a specific structural form, and then developed into a relatively fixed cultural model, forming its own unique cultural tradition. This unique cultural characteristic makes it gradually permeate into all aspects of people's lives in the continuous development and inheritance. It not only deeply affects the way of thinking and value orientation of people but also affects their psychological ecosystem. Moreover, it influences people's views on mental health by changing their cognitive styles. Therefore, it can be understood as an “invisible hand,” which unconsciously affects the psychology of people and guides people to move toward a common value orientation (Zhou, 2018). The psychological education of college students is a process of cultivating and perfecting people with purpose and plan, and its progress and development are inseparable from the definite educational content. The process of determining the content of education is also the process of choosing culture and absorbing the essence of culture. As one of the most important forms of the modern education system, the content of mental health education in college students also needs to be determined according to the local background and situation, that is, to absorb the culture with Chinese characteristics infiltrated in the national theory and discourse system.

### Influence of National Theory Discourse System on Psychological Education

The discourse system of philosophy and social sciences of any country or nation will be branded with the spirit of that country and nation. Hegel said: “only when a nation has mastered a science in its own language, can it be said that the science belongs to the nation.” For a country or a nation, discourse is the representation of external identity and internal spirit. Therefore, the construction of the pedagogy discourse system with Chinese characteristics, Chinese manner, and Chinese style has important theoretical and practical significance for breaking the discourse hegemony of Western pedagogy, establishing the discourse power of Chinese pedagogy, and promoting the “going out” of Chinese pedagogy (Zhang and Jin, 2018). Any culture can be divided into advanced and backward, appropriate and inappropriate. As a kind of guidance work with purposeful cultural value, the mental health education of college students needs to analyze and evaluate their actual psychological state according to their real psychological development needs, and provide students with scientific and valuable cultural resources after eliminating the false and retaining the true. Ideological and political classroom is high-quality culture within the scope of culture with Chinese characteristics. Its contents meet the needs of modern society and the psychological development of college students. These two factors make it inevitable for the mental health education of college students to choose and absorb the essence of the theoretical system of culture with Chinese characteristics and make it gradually evolve into an irreplaceable part of the mental health education system (Gzulle and Jin, 2018). The process of inheriting the culture with Chinese characteristics in the mental health education of college students will make the Sinicization of Marxism have new significance, and carry out the innovative application according to the current social education situation and the actual psychological situation of college students (Chen and Yu, 2018). The ethnic theory will also be combined with western psychological culture to derive more meaningful theoretical elements on the basis of the original functions, which often become a new bud in the system of Chinese ethnic theory (Rokhman and Ahamed, 2018; Zheng and Wang, 2020). The correlation test among variables reveals that there is a correlation among different grades, acceptance, and attendance. Meanwhile, the obtained α coefficient of four different dimensions is >0.7, indicating that the questionnaire has high reliability.

### Experimental Design of Questionnaire on the Acceptance of Psychological Education in College Students

College students are the “main force” of mental health education. The corresponding questionnaire was set up to understand the development status of mental health education of college students in detail. The study takes local college students as the main survey object, and uses the principle of voluntary participation to conduct a questionnaire survey on students of different majors, grades, and ages. A total of 250 students participated in the survey, 230 valid questionnaires were collected again, and the recovery rate was 92%. Then the questionnaire data are input into IBM SPSS version 22.0 (IBM Corp., Armonk, NY, USA) for analysis, and the α-coefficient of the questionnaire in four different dimensions is >0.7, indicating that the questionnaire has high reliability. The questionnaire mainly includes four aspects: (1) the discourse content of ideological and political education; (2) views of college students on the theoretical course of ideological and political education in colleges; (3) cognition of college students on ideological and political education activities of counselors; and (4) the publicity content of campus culture. The questionnaire adopts the five-point scoring method, in which 5 points, 4 points, 3 points, 2 points, and 1 point represent five attitudes: very satisfied, satisfied, average, dissatisfied, and very dissatisfied, respectively. The higher the score, the higher the ideological and political quality and psychological level of college students.

## Influence of the Discourse System on Psychological Education in College Students

### Questionnaire on Psychological Education of College Students

The correlation among the selected questionnaire research data was tested using the statistical software IBM SPSS version 22.0 (IBM Corp., Armonk, NY, USA) for data analysis. Table 1 show the specific results.

**Table 1:** Correlation test results among different variables.

**Project**	**Students of different grades**	**Acceptance of students in different grades**	**Attendance of students in different grades**
Students of different grades	0.712^**^	0.5856^**^	0.5961^**^
Acceptance of students in different grades	0.5269^**^	0.5697^**^	0.6245^**^
Attendance of students in different grades	0.634^**^	0.6893^**^	0.6358^**^

** *means significant*.

Table 1 shows the results of correlation test among students of different grades and their acceptance of psychological education course and attendance rate of the course. The results show that the correlations among students in different grades, their course acceptance, and their attendance are 0.712, 0.5856, and 0.5961, respectively, indicating that there is a positive correlation among them. Similarly, the correlations among the acceptance of psychological education courses of students, their grades, and course attendance are 0.5269, 0.5697, and 0.6245, respectively, indicating that they also have a positive correlation with each other. The correlation among the attendance rate of students in different grades on psychological education courses and the acceptance of students and courses in each grade is 0.634, 0.6893, and 0.6358, respectively. Similarly, this shows that there is also a correlation among them, and the correlation is the highest among the three groups of data. Generally, the data above 0.7 indicate a very close relationship, the data between 0.4 and 0.7 suggest the close relationship, and the data between 0.2 and 0.4 suggest a general relationship. It means that the questionnaire data selected have a good correlation among various factors, which can meet the research requirements of this exploration.

**Figure 1:**
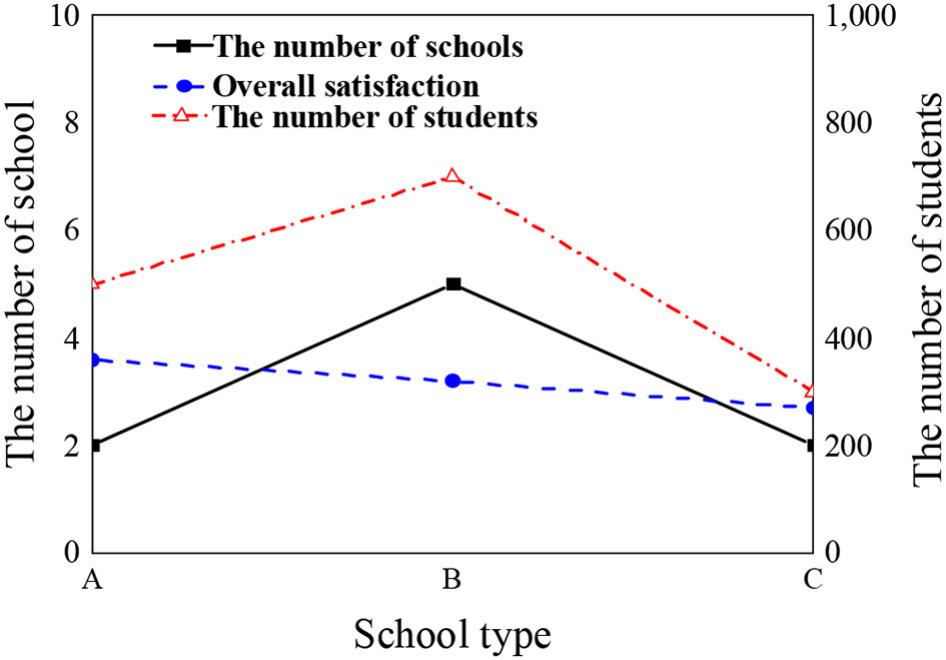
Distribution of students and overall satisfaction scores of different school types.

Figure 1 is about the distribution of school types and the number of students in the subjects of the questionnaire.

Figure 1 displays that the overall satisfaction score of students in class A colleges on mental health education is about 3.8, that in class B colleges is about 2.9, and that in class C colleges is about 2.3. It reveals that student satisfaction with mental health education will change with different colleges.

In nine local colleges, a total of 250 questionnaires are sent out, and 230 valid questionnaires are collected, with a recovery rate of 92%. Among them, class A is key colleges, class B is the general undergraduate colleges, and class C is junior colleges and higher vocational colleges. Tables 2, 3 show the distribution of gender, nationality, grade, and age of specific students.

**Table 2:** Gender distribution of students.

**Gender**	**Number of students**
Male	123
Female	107

**Table 3:** Nationality distribution of students.

**Nationality**	**Number of students**
The Han nationality	158
Uygur	15
Bourau	23
Hui	3
Manchun	16
Mongols	15

**Figure 2:**
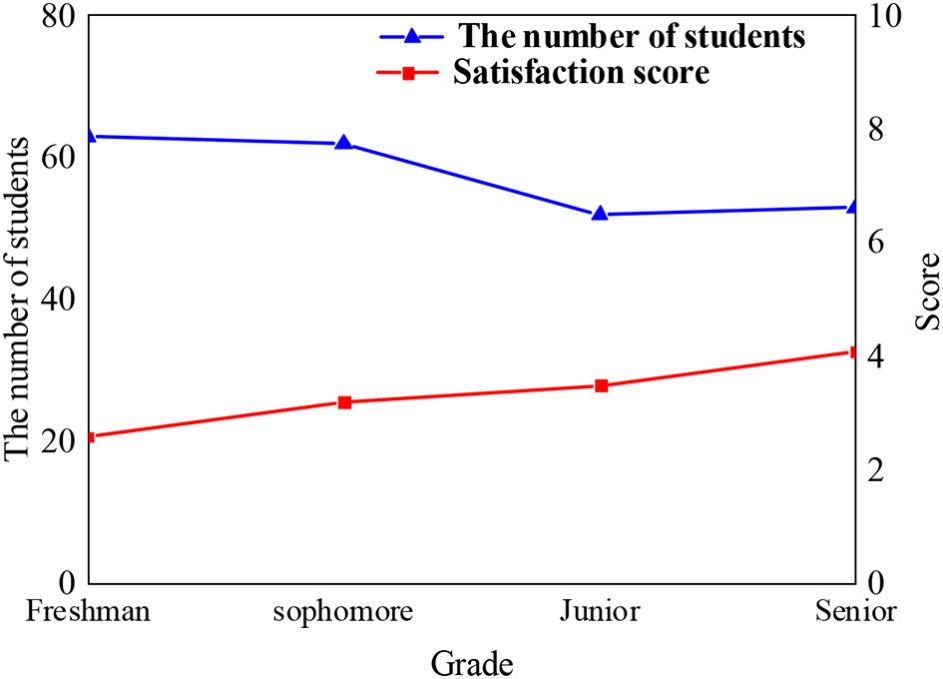
Distribution and satisfaction of students in different grades.

**Figure 3:**
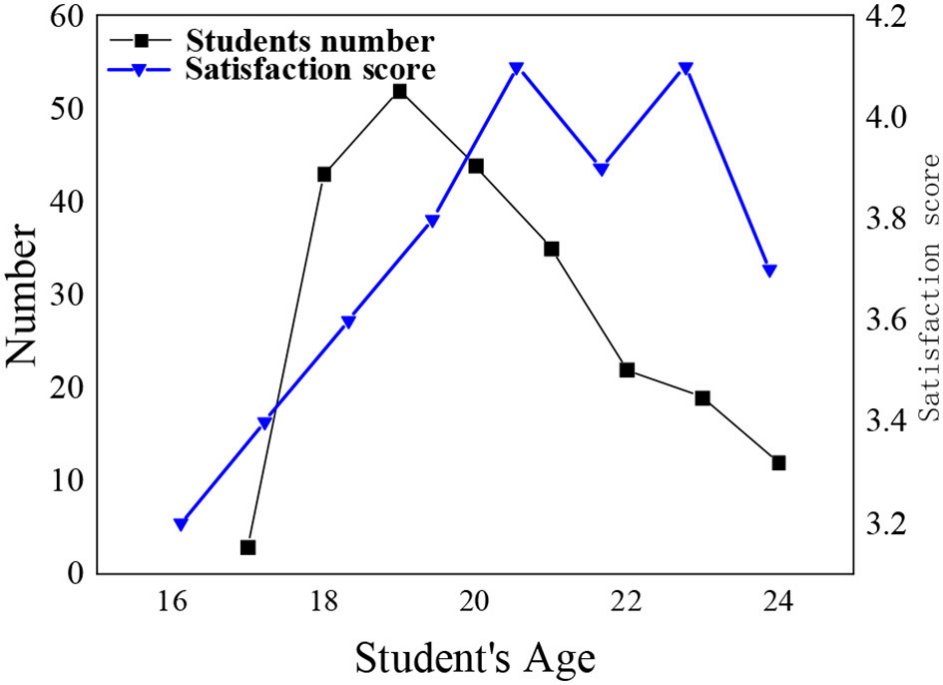
Distribution and satisfaction of students of different ages.

Discourse is a word, sentence, symbol, or information carrier that expresses the ideas, knowledge theory, emotion, and culture of a certain stage of human society. The discourse system of Chinese national theory has been in a state of aphasia for a long time, and the discourse communication carrier is single, which has brought many negative effects to production and life, reflecting the lack of confidence in Chinese national theory. In order to better distinguish the acceptance of psychological education of students, block division is performed according to grade and age. Figures 2, 3 show the distribution of students in different grades and age groups.

According to the distribution of students selected in the survey process, the proportion of male and female is relatively balanced, and the number of students in each grade has little difference. Student satisfaction with mental health education in different grades shows that student satisfaction with mental health education has been improved with the increase of grade. The age of the respondents is concentrated between 18 and 20 years old. In order to ensure the objectivity and impartiality of the survey results, the number of minorities in the surveyed students accounts for about one-third of the total number of respondents.

### Relationship Between Psychological Education and the Attendance Rate of Ideological and Political Classroom to Class

**Figure 4:**
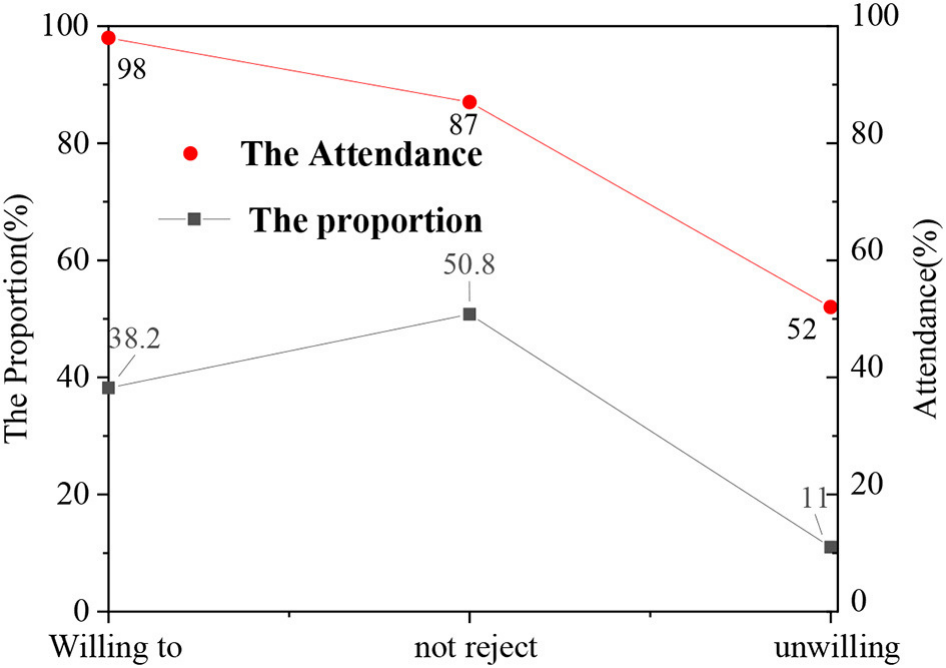
Comparison of attendance rate of ideological and political course among students with different satisfaction levels.

In the questionnaire, the acceptance of students for school psychological education is divided as: willing to, not rejected, and unwilling. Meanwhile, after the questionnaire of different levels, the attendance rate of ideological and political class is counted. Figure 4 shows the statistical results.

Figure 4 suggests that the students who actively accept psychological education also have a high attendance rate in the ideological and political class, whereas the students who refuse to accept psychological education have a low attendance rate. Ideological and political classes generally include Marxist theory, theoretical system, forms and policies of socialism with Chinese characteristics, outline of modern Chinese history, and other courses, which are important contents of the theoretical discourse system of the Chinese nation. The attendance rate of the ideological and political class can essentially reflect the recognition of students on national theory, so psychological education can help improve the enthusiasm of students for ideological and political class to a certain extent, and the discourse system of national theory provided by the ideological and political class also has an important impact on the mental health of students and national self-confidence.

### Acceptance of Psychological Education by Students of Different Grades

**Figure 5:**
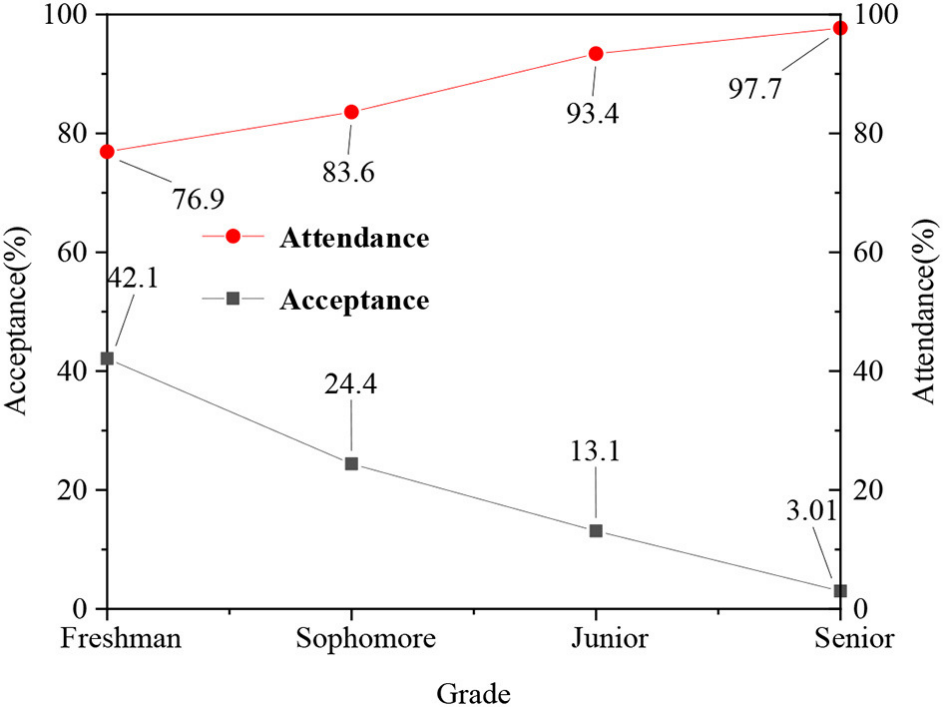
Comparison of curriculum satisfaction and attendance rate of students in different grades.

Through the statistics and arrangement of the questionnaire, the acceptance degree of psychological education and the attendance rate of ideological and political class of students in different grades are shown in Figure 5.

Figure 5 shows that for students of different grades, their willingness to accept psychological education is also different. Freshmen, as a group just entering the college campus, their personality is strong, and the willingness to accept psychological education is not very big. After the ideological and political classroom education, their acceptance increases year by year. For example, in the freshman year, the main content of ideological and political class is ideological and moral cultivation and legal basis. In the sophomore year, in the ideological and political class, teachers begin to teach Marxism, Mao Zedong Thought, and the theoretical system of socialism with Chinese characteristics. The deepening of the discourse system of national theory is also a positive factor for students to receive mental health education. The obvious improvement of the attendance rate in the ideological and political class shows that the ideological and political course plays a certain role in promoting psychological education for students.

### The Influence of Nationality and Gender on Mental Health Education

**Figure 6:**
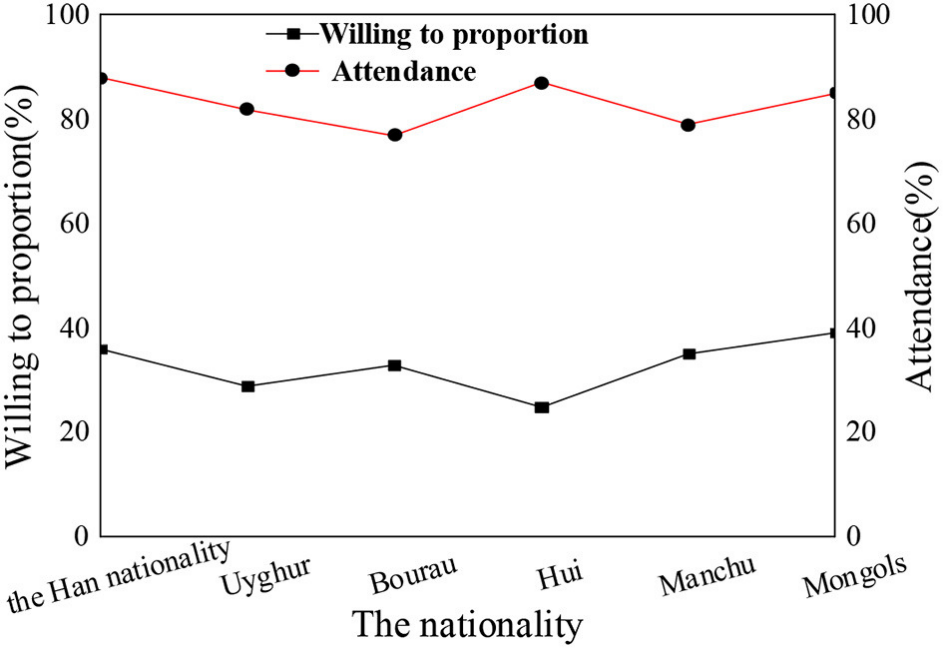
Acceptance of psychological education and attendance rate of ideological and political class among students of different nationalities.

**Figure 7:**
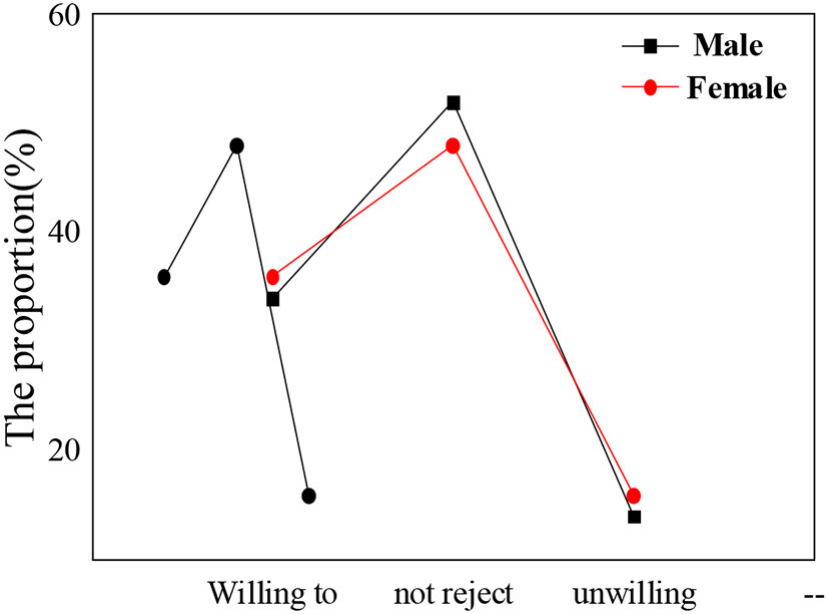
Acceptance of psychological education and attendance rate of ideological and political class among students of different genders.

**Figure 8:**
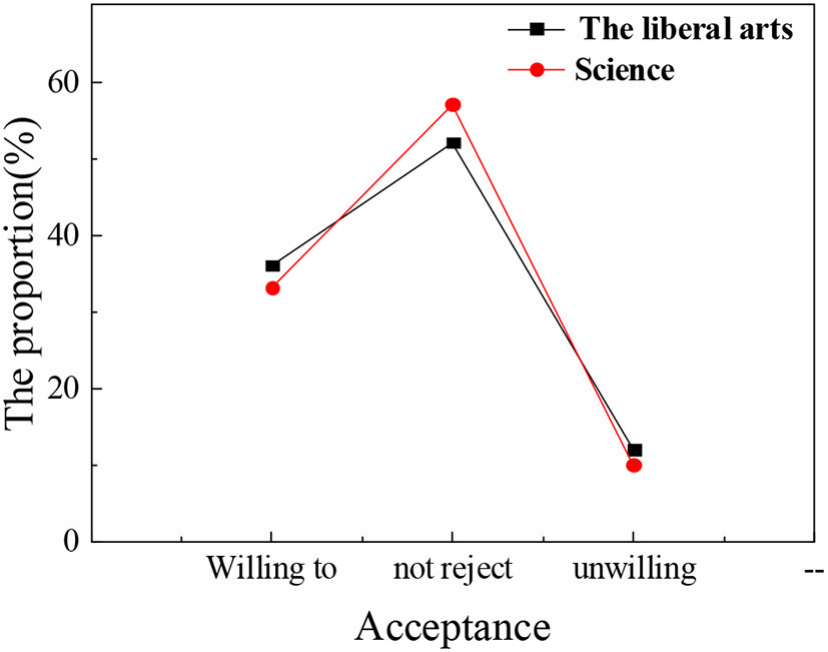
Acceptance of psychological education and attendance rate of ideological and political class among different science students.

In the questionnaire, the psychological education acceptance degree for students of different nationalities, different genders, and different subjects is statistically analyzed. Figures 6–8 show the results.

The statistical results show that the nationality of students, gender, and subject type have little influence on the acceptance of psychological education. The overall proportion of students who are willing to actively accept psychological education is about 30–35%. In the process of receiving psychological education, nationality differences in students, gender differences, and subject differences are not obvious. Compared with the results of the previous section, grade differences have the most obvious impact on the process of psychological education. There are many academic studies on the correlation between the theoretical discourse system of the Chinese nation and psychological education of college students, but they are lack of comprehensiveness and depth. The conclusions based on the attendance rate of children of different ages in psychological education classes and the acceptance and satisfaction of psychological education by different nationalities, gender, and disciplines are more persuasive and effective than the theories put forward by other scholars and have more reference significance for future research in this field.

## Conclusion

The theoretical discourse system of the Chinese nation is a summary of civilization and culture, a part of Chinese soft power, and can represent its external image. A sound and complete discourse system can enable China to fully demonstrate its strength on the international stage. In order to further promote the construction of the national discourse system in the mental health education system of Chinese college students, the current situation of the theoretical discourse system of the Chinese nation and the psychological education of college students are studied, and the correlation between them is analyzed. On the basis of integrating theory with practice, a questionnaire is conducted in nine local colleges. The results show that the difference in student satisfaction with ideological and political education in different types of schools is not very obvious, but student satisfaction with mental health education shows a trend of gradually increasing with the increase of grade. The average satisfaction of senior students reaches 4 points, indicating that senior students are most satisfied with the mental health education in school. Furthermore, the higher the satisfaction of mental health education is, the higher the attendance rate is. The attendance rate of students of different grades in ideological and political courses is also different. The attendance rate of senior students is the highest, 98%, whereas that of freshmen is the lowest, only 87%. Students willing to actively accept psychological education account for 30–35% of the total number of students. In the process of receiving psychological education, ethnic differences, gender differences, and subject differences in students are not obvious. The difference of satisfaction and attendance between different grades mainly comes from the time of receiving mental health education. Freshmen have not yet received mental health education, whereas senior students have high ideological and political literacy after 4 years of mental health education. Therefore, mental health education can be strengthened to improve the attendance rate of ideological and political courses in students, and ensure that students maintain good mental health. The education of national theory discourse system with Marxism and socialism with Chinese characteristics as the main content has a certain role in promoting psychological education of college students, and the ideological and political classroom plays an important supporting role in the establishment and development of the national theory discourse system. The shortcomings of this exploration are as follows. The psychological education of college students is a complex process, and the influencing factors are different in different periods. Due to the limited ability, only some obvious superficial factors are studied, and the internal resistance factors of the psychological education of college students are not deeply studied. In view of this defect, a single factor analysis of the population needs to be added in the follow-up investigation, and family conditions, registered residence, and whether or not the respondent is an only child should be included in the investigation. It is believed that the theoretical discourse system of the Chinese nation will be better developed in the future. The great rejuvenation of the Chinese nation will be realized step by step, and the “Chinese” discourse will be spoken to the world in its own way.

## Data Availability Statement

The raw data supporting the conclusions of this article will be made available by the authors, without undue reservation.

## Ethics Statement

The studies involving human participants were reviewed and approved by Xichang University Ethics Committee. The patients/participants provided their written informed consent to participate in this study. Written informed consent was obtained from the individual(s) for the publication of any potentially identifiable images or data included in this article.

## Author Contributions

All authors listed have made a substantial, direct, and intellectual contribution to the work and approved it for publication.

## Funding

This work was supported by the 2021 Talent Project of Xichang University through a Path-Designing Research on the Going out Strategy of Liangshan Yi Culture, under Project YBS202101, and 2021 Planning Project of Sichuan Federation of Social Sciences through an Innovation Research on the Going out Strategy of Liangshan Yi Culture, under Project SC21C060.

## Conflict of Interest

The authors declare that the research was conducted in the absence of any commercial or financial relationships that could be construed as a potential conflict of interest.

## Publisher's Note

All claims expressed in this article are solely those of the authors and do not necessarily represent those of their affiliated organizations, or those of the publisher, the editors and the reviewers. Any product that may be evaluated in this article, or claim that may be made by its manufacturer, is not guaranteed or endorsed by the publisher.
